# Myco-synthesized copper oxide nanoparticles using harnessing metabolites of endophytic fungal strain *Aspergillus* *terreus*: an insight into antibacterial, anti-*Candida*, biocompatibility, anticancer, and antioxidant activities

**DOI:** 10.1186/s12906-023-04056-y

**Published:** 2023-07-22

**Authors:** Abdel-Rahman A. Nassar, Hossam M. Atta, Mohamed Ali Abdel-Rahman, Wageih S. El Naghy, Amr Fouda

**Affiliations:** 1 Tanta Universal Teaching Hospital, Tanta University, Tanta, Egypt; 2 Botany and Microbiology Department, Faculty of Science, Al-Azhar University, Nasr City, 11884 Cairo Egypt; 3 Department of Medical Microbiology and Immunology, Faculty of Medicine, Tanta University, Tanta, Egypt

**Keywords:** Endophytic fungi, *Aspergillus terreus*, Nanoparticles biosynthesis, Antimicrobial, Anticancer, DPPH scavenging

## Abstract

**Background:**

The overuse of antibiotics leads to the emergence of antibiotic-resistant microbes which causes high mortality worldwide. Therefore, the synthesis of new active compounds has multifunctional activities are the main challenge. Nanotechnology provides a solution for this issue.

**Method:**

The endophytic fungal strain *Aspergillus terreus* BR.1 was isolated from the healthy root of *Allium sativum* and identified using internal transcribed spacer (ITS) sequence analysis. The copper oxide nanoparticles (CuO-NPs) were synthesized by harnessing the metabolites of the endophytic fungal strain. The UV-Visble spectroscopy, Fourier-transform infrared spectroscopy (FT-IR), Transmission electron micrscopy (TEM), Energy dispersive X-ray (EDX), X-ray diffraction (XRD), Dynamic light scattering (DLS), and zeta potential (ζ) were used for the characterization of synthesized CuO-NPs. The activity against different pathogenic bacteria and *Candida* species were investigated by agar well-diffusion method. The biocombatibility and anticancer activity were assessed by MTT assay method. The scavenging of DPPH was used to investigate the antioxidant activity of synthesized CuO-NPs.

**Results:**

Data showed the successful formation of crystalline nature and spherical shape CuO-NPs with sizes in the ranges of 15–55 nm. The EDX reveals that the as-formed sample contains ions of C, O, Cl, and Cu with weight percentages of 18.7, 23.82, 11.31, and 46.17%, respectively. The DLS and ζ-potential showed high homogeneity and high stability of synthesized CuO-NPs with a polydispersity index (PDI) of 0.362 and ζ-value of − 26.6 mV. The synthesized CuO-NPs exhibited promising antibacterial and anti-*Candida* activity (concentration-dependent) with minimum inhibitory concentration (MIC) values in the ranges of 25–50 µg mL^–1^. Moreover, the fungal mediated-CuO-NPs targeted cancer cells of MCF7 and PC3 at low IC_50_ concentrations of 159.2 ± 4.5 and 116.2 ± 3.6 µg mL^–1^, respectively as compared to normal cells (Vero and Wi38 with IC_50_ value of 220.6 ± 3.7 and 229.5 ± 2.1 µg mL^–1^, respectively). The biosynthesized CuO-NPs showed antioxidant activity as detected by the DPPH method with scavenging percentages of 80.5 ± 1.2% at a concentration of 1000 µg mL^–1^ and decreased to 20.4 ± 4.2% at 1.9 µg mL^–1^ as compared to ascorbic acid (control) with scavenging activity of 97.3 ± 0.2 and 37.5 ± 1.3% at the same concentrations, respectively.

**Conclusion:**

The fungal mediated-CuO-NPs exhibited promising activity and can be integrated into various biomedical and theraputic applications.

## Background

Since the 1940s, antibiotics have been routinely used not only to treat bacterial diseases but also to prevent infections in compromised immune patients and to improve and enhance livestock [1]. Unfortunately, the misuse or overuse of antibiotics leads to the evolution of antibiotic-resistant or multidrug-resistant microbes which have fatal effects on health worldwide [2]. Recently, nanotechnology science is expanding to be involved in various fields such as medicines, cosmetics, medical textiles, pharmaceuticals, food, chemical industry, environmental sciences, energy science, etc. [3–6]. These high activities could be attributed to the unique properties of nanoparticles such as surface chemistry, large surface area, high stability, sizes, shapes, and promising catalytic activity [7]. Therefore, the use of these active compounds to control the growth of pathogenic microbes and to treat cancer cells is a recent trend. Although the metal and metal oxide nanoparticles are synthesized using chemical and physical methods such as sol-gel, hydrothermal, chemical reduction, solvothermal, microwave, laser ablation, pyrolysis, and gamma-ray irradiation, it is a non eco-friendly, costly method, needs harsh conditions, and producing toxic end products such as harmful gases and vapors to human and ecosystem [8]. Therefore, green synthesis of nanoparticles (NPs) using different biological entities such as bacteria, actinomycetes, fungi, yeast, and plants are preferred due to it being environmentally friendly, cheap, easy scale-up, biocompatibile, easy to handle, avoid the production of hazardous end products, and saving energy [9, 10].

Fungi are the beneficial choice for producing various metal and metal oxide NPs either extracellularly (reduction of metal ions using metabolites present in fungal biomass filtrate) or intracellularly (entry of metal ions to fungal cells and reduction inside the cell by reducing enzymes) [11]. This finding is due to it being easy to handling, producing a large amount of biomass, characterized by high metal binding strength, high metal tolerance, and high metabolite secretions [7]. In this regard, Ag-NPs, Au-NPs, ZnO-NPs, Fe_2_O_3_-NPs, MgO-NPs, TiO_2_, etc. were produced by different fungal strains [12, 13]. Among fungi, endophytic strains which dwell within plant hosts without causing any disease symptoms to their host, are characterized by the secretion of high amounts of secondary metabolites like their host either in type or activity. These metabolites can be used to fabricate various NPs with different sizes, stability, and shapes [14].

Copper oxide nanoparticles (CuO-NPs) have gained more attention due to their unique properties such as thermal and chemical stability, low toxicity, low cost, high catalytic activity, biocompatibility when incorporated into medical fields, and high compatible with various materials to produce polymers [1, 15]. These NPs have excellent applications such as antibacterial, antifungal, antifouling, anticancer, anti-helminthic, antioxidant, antidiabetic, anti-insects, medical textiles, agriculture, gas sensors, superconductors, magnetic-resistant materials, lubricants, solar cells, lithography, wastewater treatment, and photocatalytic efficacy [16, 17]. From an economic view, CuO-NPs have cost ten-fold cheaper than Ag-NPs, as well the toxicity level of CuO-NPs is low compared to Ag-NPs when applied in biomedical and biotechnological applications [18]. For example, CuO-NPs have been shown to exhibit strong antimicrobial properties against a broad range of microorganisms, including bacteria, fungi, and viruses. This makes them a promising candidate for developing new treatments for infectious diseases, particularly in cases where conventional antibiotics have become ineffective due to the emergence of drug-resistant strains [10]. Furthermore, CuO-NPs have been found to possess antioxidant properties, which may help to reduce oxidative stress and inflammation in the body. This has led to research on their potential use in preventing and treating a variety of conditions related to oxidative stress, such as cardiovascular disease and neurodegenerative disorders [19]. Finally, there is growing interest in the potential anticancer properties of CuO-NPs. Studies have shown that they may be effective in inhibiting the growth and proliferation of cancer cells, while leaving healthy cells unharmed [3, 20]. The biosynthesis of CuO-NPs using natural compounds, such as metabolites of microorganisms, especially endophytes, has gained attention as a more sustainable and environmentally friendly approach compared to traditional chemical methods. This has further increased interest in investigating their potential therapeutic applications including antimicrobial, antioxidant, and anticancer activities to lead to the development of novel and effective treatments for a variety of diseases. Different fungal strains were used to fabricate CuO-NPs such as *Stereum hirsutum* [18], *Trichoderma harzianum* [13], *Trichoderma asperellum* [21], *Fusarium oxysporum* [22], *Aspergillus terreus* [14], *Aspergillus fumigatus* [23], and *Aspergillus terreus* [24].

In the current study, the harnessing of metabolites secreted by endophytic fungal strains isolated from healthy roots of garlic plants to fabricate CuO-NPs was investigated. The fungal strain BR.1 was identified using cultural, microscopic examination, and molecular identification based on ITS sequence analysis. The as-formed NPs were characterized by color change, UV-Visble spectroscopy, Fourier-transform infrared spectroscopy (FT-IR), Transmission electron micrscopy (TEM), Energy dispersive X-ray (EDX), X-ray diffraction (XRD), Dynamic light scattering (DLS), and zeta potential (ζ). Moreover, their potential for biomedical applications including antibacterial activity, anti-*Candida* activity, biocompatibility, anticancer, and antioxidant activity were evaluated.

## Materials and methods

### Fungal isolation and identification

The fungal strain coded as BR.1 was isolated from the healthy root of *Allium sativum* L. Our work complies with the institutional, national, and international guidelines and legislation. Garlic is common worldwide and does not need permission or licenses as the species we are working with is a cosmopolitan crop that is not at risk or endemic according to IUCN. The root samples were collected from agricultural land in El-Menofia governorate, Egypt (30°38’40.9"N 30°56’49.9"E) under a permission (number: EM2/2022) from local agricultural office in the governorate. The isolation procedure was achieved according to Khalil et al. [25]. Briefly, the collected plant samples were washed with tap water to remove the attached particles. After that, the root samples were subjected to surface sterilization as follows: washing first with sterilized H_2_O for one minute followed by rinsed with sodium hypochlorite (2.5%) for 4 min, ethanol (70%) for half minutes, and finally immersed into sterilized H_2_O for two minutes. The successful surface root sterilization was confirmed by inoculation of an agar plate (nutrient for bacteria, starch nitrate for actinomycetes, and Czapek Dox for fungi) by final washing H_2_O. The absence of any bacterial, actinomycetes, or fungal growth confirmed the successful surface sterilization process.

Approximately, five segments (4 mm/segment) of sterilized garlic roots were loaded on the surface of a potato dextrose (PD) agar (Ready-prepared, Oxoid) plate containing chloramphenicol as an antibacterial agent and incubated for 7 days at 25 ± 2 °C. The loaded plates were observed daily to picked-up the fungal colony originating from internal plant tissues and re-inoculation into new PD agar plate for purification.

The selected fungal strain designated as BR.1 was subjected to traditional identification based on morphological and microscopic examination according to the standard method [26], followed by molecular identification based on ITS sequence analysis. The amplifications and sequencing of the ITS gene were accomplished according to White et al. [27] using a primer of ITS1 (5´ CTTGGTCATTTAGAGGAAGTAA-3´) and ITS4 (5´ TCCTCCGCTTATT GATATGC 3´). The PCR protocol and sequencing method was achieved as mentioned previously [28]. The sequence of the ITS gene was compared by those deposited in GenBank using ClustalX 1.8 software package (http://www.clustal.org/clustal2). Moreover, the phylogenetic tree was accomplished by the neighbor-joining method (MEGA v6.1) software, with confidence tested by bootstrap analysis (1000 repeats).

### Biosynthesis of CuO-NPs using cell-free filtrate of BR.1

The endophytic fungal strain BR.1 was inoculated into 100 mL of PD broth media and incubated at 25 ± 2 °C for 5 days. At the end of the incubation period, the inoculated PD broth media (Ready-prepared, Oxoid) was centrifuged at 10,000 rpm for 5 min to collect the fungal biomass which was rinsed thrice with dis. H_2_O to remove any medium component adhering. After that, 10 g of collected fungal biomass was mixed with 100 mL dis. H_2_O for 24 h. in dark conditions under a shaking state (150 rpm). Thereafter, the mixture was centrifuged to collect the supernatant (fungal biomass filtrate) to use as a biocatalyst for the biosynthesis of CuO-NPs.

The components of fungal biomass filtrate were analyzed using Gas Chromatography–Mass Spectrometry (GC-MS, Agilent Technologies, Santa Clara, CA, USA) which used gas chromatography (7890B) and mass spectrometer detector (5977 A). The column used in GC system was HP-5MS which have a diameter of 30 m x 0.25 mm, film thickness of 0.25 m, and gas carrier was hydrogen at a flow rate of 1 mL/min. The temperature during analysis was adjusted as follows: 50 °C hold for one minute, followed by a 5 °C/minute increase to 100 °C, and finally a 10 °C/min increase to 300 °C for five minutes. The injector and detector temperatures were also tweaked to 250 and 260 °C respectively. Mass spectra were generated between 50 and 550 m/z with an electron ionization energy of 70 eV and a solvent delay of six minutes [29]. By comparing the acquired fragmentation chart to those stored in the Wiley and NIST Mass Spectral Library [30], the contents of biomass filtrate were determined.

The biosynthesis of CuO-NPs using fungal biomass filtrate was acheived as follows: 100 µg of Cu(CH_3_COO)_2_‧H_2_O (98%, Sigma Aldrich, cairo, Egypt) was dissolved in 5 mL dis. H_2_O before being mixed with 95 mL fungal biomass filtrate to get a concentration of 5 mM. The final mixture was stirred for 1 h. at 100 rpm and adjusted the pH at 8 by adding 1 N NaOH drop-wisely [13]. Simultaneously, the fungal biomass filtrate without metal precursor was used as a positive control. The color change of biomass filtrate from colorless to greenish indicates the formation of CuO-NPs. The as-formed NPs were collected by centrifugation at 10,000 rpm for 10 min and washed thrice with dis. H_2_O and subjected to calcination at 200 °C for 2 h. before being collected in Eppendorf and preserved at room temperature.

### Characterization

#### UV-Vis spectroscopy

The intensity of the greenish color that formed after mixing fungal biomass filtrate with metal precursor was checked using UV-Vis spectroscopy (JENWAY 6305, Staffordshire, UK) to measure the absorbance at a wavelength in the range of 200–800 nm. In total, a quartz cuvette was filled with 2 mL of synthesized solution and measured their absorbance at regular intervals time to investigate the maximum surface plasmon resonance (SPR) [16].

#### FT-IR

The functional groups in fungal biomass filtrate and compared with the functional groups in biosynthesized CuO-NPs were investigated by FT-IR (Agilent system, Cary-660 model). In this method, 10 mg of biosynthesized CuO-NPs was mixed with potassium bromide (KBr, ≥ 99.0%), mixed well, and pressed under pressure to form a disk before being scanned at wavenumbers in the ranges of 400–4000 cm^–1^ [31].

#### TEM and EDX

The morphological characteristics including shapes and sizes of fungal-mediated biosynthesis of CuO-NPs were assessed by Transmission electron microscopy (TEM, JEOL, Ltd-1010, Tokyo, Japan). In this analysis, the powder of CuO-NPs was scattered in high-purity solvent (MiliQ H_2_O) by ultra-sonification followed by a loaded few drops of this suspension on a carbon grid. The loaded grid was touched with the blotting paper to remove the excess solution before being subjected to scanning [32]. The elementary mapping including the qualitative and quantitative composition of CuO-NPs was investigated by Energy dispersive X-ray (EDX) apparatus (JEOL, JSM-6360LA, Tokyo, Japan) through scanning electron microscopy image analysis.

#### XRD

The nature phase (crystalline or amorphous) of biosynthesized CuO-NPs was examined by X-ray diffraction (PANanalytical-X’Pert-Pro-MRD, Philips, Eindhoven, the Netherlands) connected with Cu-Kα as X-ray source (λ = 1.54 Å) at a voltage and current of 40 KV and 30 mA respectively. The X-ray scanning was achieved in the range of two Theta values from 10° − 80°. The average crystallite size of CuO-NPs was calculated according to XRD analysis by Debye–Scherrer’s equation as follows [33]:

1 $$\mathrm L=\frac{0.94\times1.54}{\upbeta\mathrm{cos}\uptheta}\times100$$

Where L is the average crystallite size; 0.94 is a Scherrer constant, 1.54 is a λmax of X-ray, β, and θ are the full width of the peak at a half maximum and diffraction angle respectively.

#### DLS and ζ-potential analysis

The distribution and hydrodynamic sizes in the colloidal solution were analyzed by dynamic light scattering (DLS) (Nano-ZS, Malvern Ltd, Malvern, UK). The synthesized CuO-NPs were suspended in MiliQ H_2_O to prevent the appearance of additional shadow on the signal during analysis. Moreover, the surface charge of synthesized CuO-NPs was detected by the Zetasizer apparatus (Nano-ZS, Malvern, UK) [34].

### Antimicrobial activity

The antimicrobial activity of fungal synthesized CuO-NPs was evaluated toward varied pathogenic microbes including Gram-positive bacteria (*Bacillus subtilis* ATCC6633, and *Staphylococcus aureus* ATCC6538), Gram-negative bacteria (*Escherichia coli* ATCC8739, *Pseudomonas aeruginosa* ATCC9027), and unicellular fungi represented by various *Candida* species (*C. glabrata, C. albicans, C. parapsilosis*, and *C. tropicalis*). The *Candida* species were clinical species isolated previously from pregnant women and identified using traditional and molecular methods in Microbiology Laboratory, National Research Centre, Giza, Egypt. The antimicrobial activity was investigated using the agar well diffusion method [35]. At the first, the pathogenic microbes were subcultured on nutrient agar (containing g L^–1^: peptone, 5; beef extract, 3; NacL, 5, Agar, 15, dis. H_2_O, 1 L) and sabouraud dextrose agar (Ready-prepared, Oxoid) plates for bacterial and *Candida* species respectively for 24 h and incubated at 35 ± 2 °C. A single colony from each strain was picked up using a sterilized swab and reinoculated uniformly on the surface of the Muller-Hinton agar plate (Ready prepared, Oxoid) followed by making wells (4 wells (0.6 mm in diameter/plate). Different concentrations (400, 300, 200, 100, 50, 25, and 12.5 µg mL^–1^) were prepared, and add 100 µL from each concentration was to the well. The DMSO (solvent, ≥ 99.7%, Merck, Germany) was added to the well and ran as a control. The loaded plates were kept in the refrigerator for 1.0 h. before being incubated at 35 ± 2 °C for 24 h. and recorded the results which were represented by the clear zone (mm) formed around each well [3]. The lowest CuO-NPs concentration that forms the inhibition zone around the well is represented as minimum inhibitory concentration (MIC value). The experiment was achieved in triplicate.

### Biocompatibility and In-vitro cytotoxicity

The cytotoxic efficacy and biocompatibility of green synthesized CuO-NPs were investigated against two cancer cells MCF7 (human breast cancer), PC3 (prostate cancer cell), and two normal cells, Vero (monkey kidney epithelial cell), and WI38 (Human lung fibroblast). These cell lines were obtained from the Holding Company for Biological Products and Vaccines (VACSERA), Cairo, Egypt. The MTT (3-(4,5-dimethylthiazol-2-yl)-2,5-diphenyl tetrazolium bromide) assay method was used to evaluate the toxicity of CuO-NPs against cancer and normal cells. Each cell type was inoculated in 96-well tissue culture plates with intensity 1 × 10^5^ cells/100 µL/well and incubated at 37 °C for 24 h in a 5% CO_2_ incubator. Once the monolayer sheet was formed, it was rinsed twice with washing media and adding 100 µL of Roswell Park Memorial Institute (RPMI-1640) maintenance media (Sigma-Aldrich, USA) with 2% serum. After that, the growing cells were treated with double-fold concentrations of CuO-NPs (1000–31.25 µg mL^–1^) and incubated for 48 h [15]. Three wells without CuO-NPs were used as a control. After the incubation period, the remaining media in each well was discarded and received 50 µL of MTT solution (5 mg mL^–1^ of phosphate buffer saline solution) and shaken thoroughly for 5 min before being incubated for 4 h. at 37 °C. After the complete incubation period, the MTT solution was discarded, and adding 100 µL of DMSO (10%) was to dissolve the formed formazan crystal through shaking for 30 min [36]. The absorbance of the formed color was measured at 570 nm by an ELIZA reader. The cell viability percentages were calculated by the following equation:

2 $$\mathrm{Cell}\;\mathrm{viability}\;\left(\%\right)=\frac{\mathrm{Absorbance}\;\mathrm{of}\;\mathrm{treatment}}{\mathrm{Absorbance}\;\mathrm{of}\;\mathrm{control}}\times100$$

### Antioxidant activity

The antioxidant activity of biosynthesized CuO-NPs was assessed using DPPH (2,2-diphenyl-1-picrylhydrazyl, Sigma-Aldrich, USA) method. Briefly, various concentrations of green synthesized CuO-NPs (1000–1.95 µg mL^–1^) were prepared by dissolving in high pure water (Milli-Q H_2_O). After that, one mL of the prepared solution was added to a test tube containing one mL of DPPH (prepared in methanol) and 450 µL of Tris-HCl buffer (pH 7.4, 50 mM), mixed well, and incubated at 37 °C for 30 min. under shaking (100 rpm) in dark condition. Another set of experiment using ascorbic acid (positive control) was achieved under the same conditions and the same concentrations. Also, the negative control which was DPPH and Tris-HCl buffer in absence of CuO-NPs or ascorbic acid was running with the experiment under the same incubation conditions. At the end of the incubation period, the absorbance of the formed color was measured at 517 nm. The free radical scavenging activity was calculated using the following equations [37]:

3 $$\begin{array}{c}\mathrm{DPPH}\;\mathrm{scavenging}\;\mathrm{activity}\;\left(\%\right)=\\\frac{\mathrm{Absorbance}\;\mathrm{of}\;\mathrm{control}\;-\;\mathrm{Absorbance}\;\mathrm{of}\;\mathrm{treatment}}{\mathrm{Absorbance}\;\mathrm{of}\;\mathrm{control}}\times100\end{array}$$

Where control and treatment are represented by ascorbic acid and CuO-NPs, respectively.

### Statistical analysis

The obtained data were statistically analyzed using package SPSS v17 and the data were represented by the means of three independent replicates. The t-test or ANOVA followed by the Tukey HSD test at *p <* 0.05 was used to measure the difference between treatments.

## Results and discussion

### Endophytic fungi for the biosynthesis of CuO-NPs

Recently, metal and metal oxide NPs are incorporated into several goods such as antimicrobial agents, antivirals, antioxidant agents, wound dressing, medical clothes, insecticides, agricultural products, filters for water and wastewater treatment, and heavy metal removals [38–41]. Therefore, great efforts are performed to synthesize these materials with rapid, eco-friendly, cost-effective, and biocompatible approaches. Green synthesis of NPs using microbes, especially endophytic strains is preferred due to the huge metabolites that are used for the reduction of metal and metal oxides to nanostructure followed by capping and stabilizing products for long times [42, 43]. In addition, the synthesis of NPs using these methods overcomes the problems that formed due to using chemical and physical approaches [7]. Among endophytic microbes, fungal strains are characterized by different metabolites that improve the NP’s stability. Moreover, fungi are preferred over bacteria, actinomycetes, and yeasts due to their easy handling, metal accumulation tolerance, high extracellular metabolite secretion, easy scale-up, and high biomass yield [44].

**Fig. 1:**
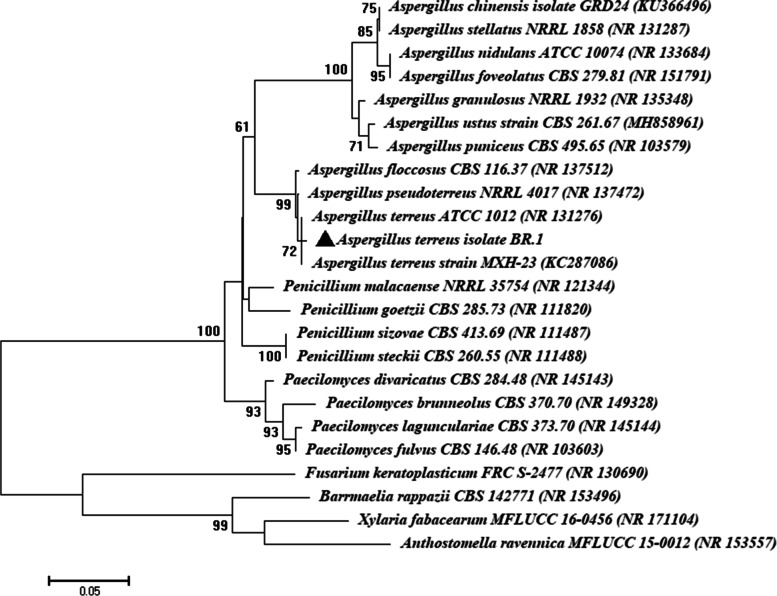
Phylogenetic tree of selected endophytic fungal strain BR.1 according to ITS sequence analysis compared to genes deposited in NCBI. The tree was constructed by MEGA-6 software via a neighbor-joining method

In the current study, endophytic fungi associated with the healthy root of garlic were isolated and investigate their efficacy in reducing, capping, and stabilizing the metal oxide precursor (Cu(CH_3_COO)_2_‧H_2_O) to form CuO-NPs. Amongst four endophytic fungal strains isolated from garlic roots, BR.1 was selected based on greenish color intensity after mixing fungal biomass filtrate with metal oxide precursor. This strain was identified based on cultural and microscopic examination as *Aspergillus* sp. followed by confirmed identification by ITS gene sequence. The molecular identification reveal that the endophytic fungal strain BR.1 was similar to *Aspergillus terreus* (closet accession number: NR131276) with a similarity percentage of 99.12%. Therefore, the selected endophytic fungal strain was identified as *A. terreus* BR.1 (Fig. 1) and the obtained sequence was deposited in GenBank under the accession number OP471233.

**Fig. 2:**
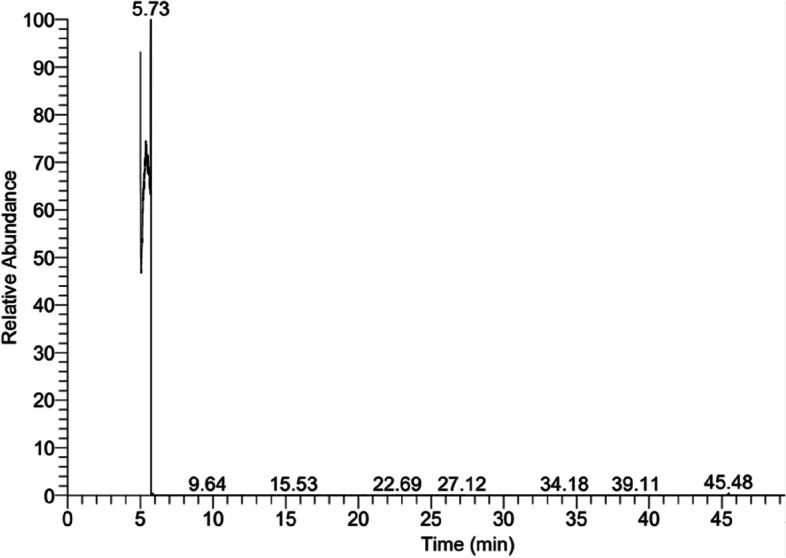
GC-MS analysis for fungal biomass filtrate showed compounds at different retention times

The CuO-NPs were synthesized by various fungal strains isolated from different sources including soils and water, but the fabrication using endophytic fungi, especially those isolated from medicinal plants will exhibit maximum bioactivities, particularly in biomedical fields. This phenomenon could be attributed to the endophytic fungi that are associated with medicinal plants has the potential to display high medicinal activities and secretion of huge bioactive substances as it mimics the activities and metabolites of the host plant [45]. Recently, CuO-NPs were synthesized by harnessing active metabolites of *A. terreus* and showed promising activity in cancer therapy [24]. In a similar study, *A. terreus* as an endophytic strain was isolated from the medicinal plant of *Aegle marmelosa* and subjected to identification based on traditional methods and ITS sequence analysis. This endophytic fungal strain was utilized to fabricate CuO-NPs which showed different biological activities including antibacterial, antifungal, antioxidant, and in-vitro cytotoxic efficacy [14]. *A. terreus* is a fungus that can produce a range of secondary metabolites, including enzymes and bioactive compounds, that have potential for use in biotechnological applications. The components of *A. terreus* BR.1 were detected using GC-MS as shown in Fig. 2; Table 1. As shown the biomass filtrate of *A. terreus* BR.1 containing various active compounds such as acids, alkenes, sugars, macrolides, and others active compounds which have a role in reduction, capping, and stabilizing final product (CuO-NPs).

The biomass filtrate of this fungus is used in the biosynthesis of wide range of nanoparticles [14, 24, 44]. The main components of the biomass filtrate of *A. terreus* that are involved in the biosynthesis of nanoparticles include proteins and enzymes, carbohydrates, organic acids, and secondary metabolites [8, 42]. For example, enzymes such as nitrate reductase and laccase can reduce metal ions to form nanoparticles, while proteins such as albumin can stabilize the nanoparticles and prevent them from aggregating [46]. Carbohydrates such as glucose and fructose are present in the biomass filtrate and can serve as reducing agents for the biosynthesis of nanoparticles [47]. *A. terreus* also produces organic acids such as citric acid and oxalic acid, which can act as reducing agents and stabilizing agents during the biosynthesis of nanoparticles [14]. Finally, secondary metabolites such as terpenoids and alkaloids can potentially act as reducing agents or capping agents during the biosynthesis of nanoparticles [7]. In the current study, the metabolites secreted by endophytic *A. terreus* BR.1 such as proteins, enzymes, and amino acids were used as a reducing agent for metal precursor (Cu(CH_3_COO)_2_‧H_2_O) to form nanoscale structure followed by capping and stabilizing the as-formed final product. The first indicator for the biosynthesis of CuO-NPs was a change in the color of fungal biomass filtrate after mixing with metal from colorless to greenish. In contrast, the color of the positive control (biomass filtrate without metal precursor) does not exhibit any change at the end of the experiment. The intensity of the greenish color is related to the percentages of reducing metal ions via fungal metabolites, the deep color indicates the complete reduction of all Cu^2+^ ions to Cu^o^ as reported previously [37].

**Table 1:** Different compounds present in fungal biomass filtrate at different retention times with their probability, area percentages (%), molecular weight (M.W.), and chemical formula based on GC-MS analysis

No.	Compounds	RT	Probability	Area %	M.W	Formula	Library
1	2-Methyl-3-pentanol	5.0	17.41	0.01	102	C_6_H_14_O	nist_ms ms
2	3-Nitro-5-methyl-2-cyanomethylpyridine	5.11	45.49	0.01	177	C_8_H_7_N_3_O_2_	Wiley9
3	Ethyl pyruvate	5.73	34.64	99.63	116	C_5_H_8_O_3_	Wiley9
4	Peracetic Acid	5.87	24.96	0.06	76	C_2_H_4_O_3_	Wiley9
5	DL-Arabinose	6.10	20.06	0.01	150	C_5_H_10_O_5_	mainlib
6	Acetic acid, 2-methylpropyl ester (CAS)	6.69	13.66	0.05	116	C_6_H_12_O_2_	Wiley9
7	Lycoxanthin	10.09	57.26	0.01	552	C_40_H_56_O	Wiley9
8	Penitrem A	14.23	5.54	0.01	633	C_37_H_44_ClNO_6_	Wiley9
9	Milbemycin b	15.53	6.07	0.01	603	C_33_H_46_ClNO_7_	mainlib
10	Russuphelol	22.69	85.23	0.01	666	C_26_H_16_Cl_6_O_8_	Wiley9
11	Monohydrokelevan	34.18	6.21	0.01	596	C_17_H_13_Cl_9_O_4_	Wiley9
12	2,5-Dibromo-1,4-di-n-hexadecylbenzene	39.11	54.37	0.01	682	C_38_H_68_Br_2_	Wiley9
13	Folic Acid	39.2	11.28	0.01	441	C_19_H_19_N_7_O_6_	Wiley9
14	4-Methoxy-5 H-pyrrolo [3,2-d]pyrimidine	45.48	39.95	0.13	149	C_7_H_7_N_3_O	Wiley9

### Characterizations

#### UV-Vis spectroscopy

**Fig. 3:**
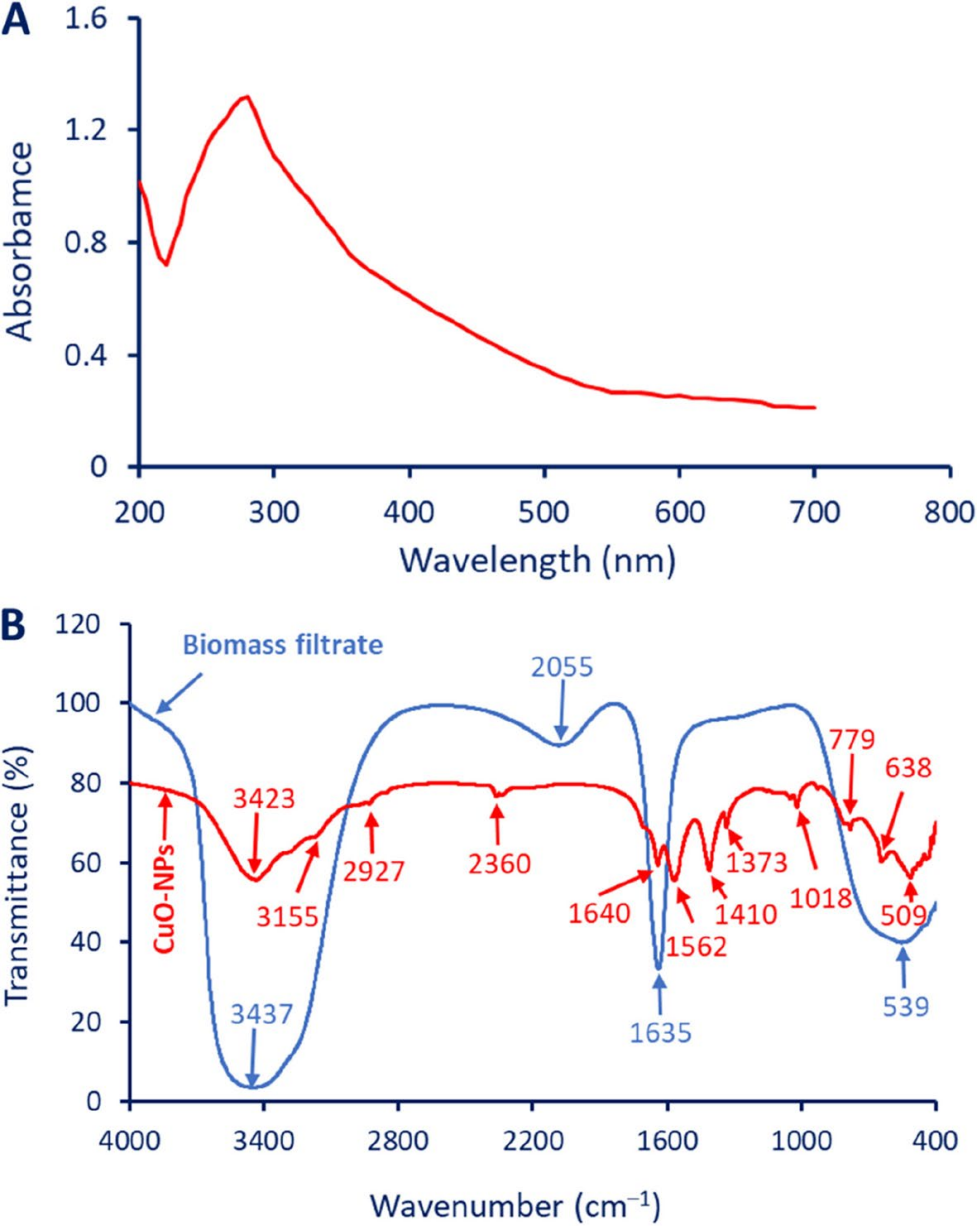
A is UV-vis spectroscopy of CuO-NPs fabricated by endophytic fungal strain *A. terreus* BR.1 showing maximum SPR at a wavelength of 280 nm; B is the FT-IR of fungal biomass filtrate versus biosynthesized CuO-NPs showing various functional groups

The intensity of the greenish color was measured using a spectrophotometer at varied wavenumbers in the ranges of 200–700 nm to detect the maximum surface plasmon resonance (SPR). As shown the maximum peak according to the UV-Vis chart was observed at a wavenumber of 280 nm which signify to the SPR of CuO (Fig. 3A). Compatible with the obtained result, the maximum SPR of CuO-NPs fabricated by *Trichoderma asperellum* was observed at a wavenumber of 285 nm [48]. In a recent study, the SPR absorption peak of CuO-NPs formed due to the harnessing metabolites of endophytic bacteria, *Brevibacillus brevis* strain PI-5 was observed at 290 nm [49]. Unfortunately, the maximum SPR peak of CuO-NPs synthesized by cell-free filtrate of endophytic *A. terreus* was shown at 551 nm [14]. The SPR absorption peak of CuO-NPs can be affected by varied factors such as crystallite size, crystallinity, shape, size, metal precursors and their concentration, and agglomeration [50]. Jayakumarai, et al. reported that the presence of one absorption peak in UV-Vis spectroscopy analysis at a wavelength in the ranges of 200–300 nm indicates the spherical shape of CuO-NPs with small sizes [51].

#### FT-IR

The role of various functional groups exists in fungal biomass filtrate in the reduction and capping of the as-formed final product as well as the new peaks related to new functional groups in synthesized CuO-NPs were investigated by FT-IR (Fig. 3B). The biomass filtrate contains four absorption peaks at a wavenumber of 3437, 2055, 1635, and 539 cm^–1^. The strong and broad peak at 3437 cm^–1^ is corresponding to the O–H stretching of hydroxyl groups overlapped with the N–H stretching of aliphatic primary amines [52]. This peak was shifted to 3423 cm^–1^ after the fabrication of CuO-NPs. The peak at 2055 cm^–1^ could be related to aromatic compound overlapping with ν(C-O) or related to CO stretching of carboxylic compounds and unsaturated ester [53, 54]. The strong peak at a wavenumber of 1635 cm^–1^ is signifying to the C═O stretching of polysaccharides or signify to I and II amide of proteins [55, 56]. This peak was shifted to a wavenumber of 1640 and 1562 cm^–1^ after the synthesis of CuO-NPs. The broadness peak at 539 cm^–1^ is related to C-S, C = S (overlapped) [56]. The weak peak at 3155 cm^–1^ is related to the C–H stretching of alkene, whereas the medium peak at 2927 cm^–1^ corresponds to the C–H stretching of alkane [53, 57]. On the other hand, the medium peak at 2360 cm^–1^ is corresponding to the C ≡ C stretching of alkyne or adsorption of CO_2_ on the surface of CuO-NPs [58, 59]. The peak at 1410 cm^–1^ signifies the bending O–H of carboxylic acid whereas the weak peak at 1373 and 1018 cm^–1^ could be attributed to the bending O–H of phenol and stretching C–N of amines respectively [37, 60]. The successful formation of Cu–O was confirmed through the presence of peaks at wavenumbers in the ranges of 500–700 cm^–1^ as reported previously [13, 33]. The presence of different functional groups related to polysaccharides, proteins, amino acids, and carboxylic acids in fungal biomass filtrate reflect their role in reduction of metal precursor to form CuO-NPs followed by capping to increase their stability.

#### Morphological characteristics

**Fig. 4:**
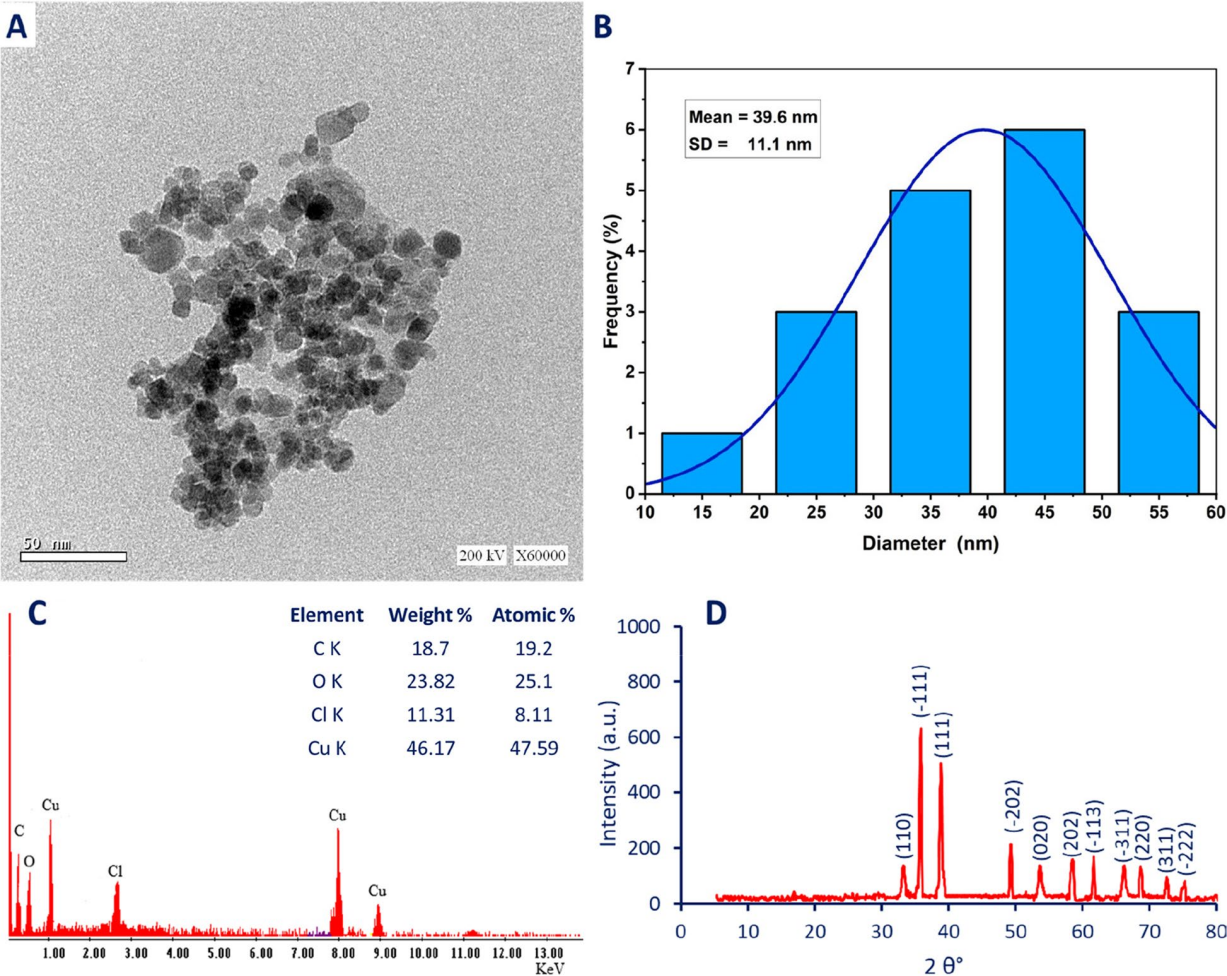
Morphological and crystallographic investigation of CuO-NPs fabricated by endophytic fungal strain *A. terreus* BR.1. **A** is Transmission Electron Microscopy (TEM) showing spherical shape, **B** is size distribution, **C** is EDX analysis, and **D** is X-ray diffraction showing crystalline nature

Transmission electron microscopy (TEM) and Energy dispersive X-ray (EDX) are the most probable techniques used for the detection of the morphological characteristics such as shape, sizes, and elemental compositions of synthesized nanomaterials [61]. Figure 4 A showed that the fungal-mediated CuO-NPs synthesis has a spherical shape and is well-arranged. Also, the synthesized CuO-NPs have sizes in the ranges of 15–55 nm with average sizes of 39.6 ± 11.1 nm (Fig. 4B). In a similar study, the sizes of CuO-NPs synthesized by endophytic fungal strain *A. terreus* isolated from *Aegle marmelosa* was less than 100 nm [14]. Also, spherical CuO-NPs were fabricated using cell-free filtrate of fungal strain *Trichoderma asperellum* with an average size of 22 nm [21]. As reported previously, the activity of nanomaterials is dependent on shape and size. For instance, the phyto-synthesized CuO-NPs using aqueous extract of *Aloe vera* with shapes of rods and platelets showed high antibacterial activity toward Gram-positive bacteria (*Staphylococcus aureus*) and Gram-negative bacteria (*E. coli*) compared to spherical shapes [62]. This activity could be attributed to the active sites on the NPs surface as well as the surface energy which differ according to structural morphologies [63]. Also, the toxicity of smaller sizes was higher than those of large sizes because of the high efficacy of small sizes to liberate toxic ions such as Cu^2+^ faster than large sizes [7, 64].

The elementary mapping of as-formed CuO-NPs was assessed by EDX. As shown the main components of the synthesized sample were Cu and O ions in addition to the presence of additional peaks for C and Cl (Fig. 4C). The absorption peak at bending energy of 0.5 KeV represented O ion whereas the peaks at bending energies of 1, 8, and 9 KeV were corresponding to Cu ions. Moreover, the absorption peaks at bending energies of 0.3 and 2.6 KeV were represented by C and Cl respectively. The weight and atomic percentages for the presence ions were (18.7, 23.82, 11.31, and 46.17%) and (19.2, 25.1, 8.11, and 47.59%) for C, O, Cl, and Cu respectively. Similarly, the weight and atomic percentages of Cu and O ions in as-formed CuO-NPs synthesized by *Trichoderma asperellum* were (79.9 and 20.04%) and (50.1 and 49.9%) respectively [21]. The authors reported that the optical absorption peaks were recorded at bending energy of 1 and 9 KeV whereas the peak at 8 KeV was referred to as the SPR of crystallite CuO. Also, the main components of green synthesized CuO-NPs were Cu and O with weight percentages of 81.4 and 18.6% respectively [65]. In the current study, the presence of C and Cl could be attributed to the scattering of capping agents from the fungal extract that coated the CuO-NPs surface [20, 66].

#### Crystallographic investigation

The crystalline nature of endophytic fungal strain-based CuO-NPs was assessed using an X-ray diffraction (XRD) pattern. As shown, there are eleven Bragg’s diffraction peaks at planes of (110), (-111), (111), (-202), (020), (202), (-113), (-311), (220), (311), and (-222) corresponding to 2θ of 33.2°, 35.7°, 38.8°, 49.2°, 53.8°, 58.7°, 61.6°, 66.4°, 68.5°, 72.6°, and 75.3° respectively (Fig. 4D). The obtained pattern confirmed that the fungal mediated-CuO-NPs have a face-centered cubic (FCC) phase with the crystalline structure according to the standard (Joint Committee on Powder Diffraction (JCPD) 80-1268). The obtained results are compatible with various published literature for green synthesis of CuO-NPs [33, 49, 67]. The diffraction peaks at 2θ values of 35° − 39° indicate the activity of fungal metabolites to fabricate CuO-NPs [68]. Moreover, the presence of well-defined and sharp XRD peaks confirmed the synthesis of the FCC crystalline phase of CuO-NPs with sizes less than 100 nm [69]. According to XRD analysis, can be concluded that the as-formed CuO-NPs were pure due to the observed sharp pattern and absence of additional diffraction peaks [21]. The average crystallite size of synthesized CuO-NPs can be calculated based on XRD analysis by Debye–Scherrer’s equation. Herein, the average crystallite size of CuO-NPs was 31 nm based on β values (full-width half maxima (FWHM)) of the sharpest peak. Incompatible with the obtained results, the crystallite size of CuO-NPs synthesized by waste fish of *labeo rohita* according to XRD analysis was 87 nm [33]. Also, the crystallite size of CuO-NPs synthesized by the fungal strain, *T. asperellum* based on FWHM of the sharpest XRD peak was 17.5 nm [21].

#### Dynamic light scattering (DLS)

**Fig. 5:**
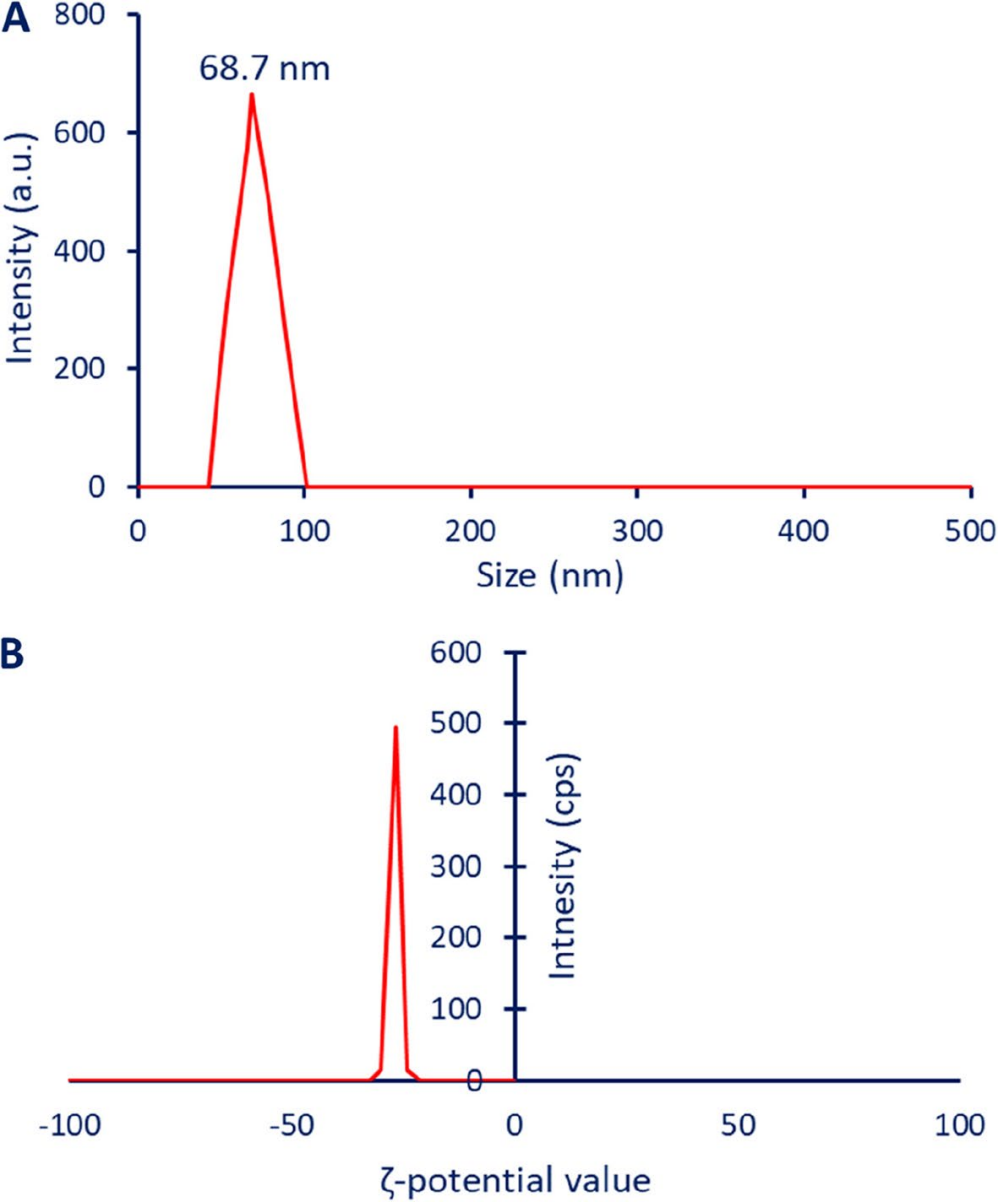
The dynamic light scattering (**A**) and zeta potential analysis (**B**) of synthesized CuO-NPs using endophytic fungal strain, *A. terreus* BR.1

The size distribution of CuO-NPs synthesized by the action of metabolites secreted by endophytic fungal strain *A. terreus* BR.1 in colloidal solution was investigated by DLS analysis. Figure 5 A showed that the average particle size of CuO-NPs after being suspended in a high-purity solvent was 68.7 nm. Comparing the average size obtained by different techniques (DLS, TEM, and XRD), can be affirmed that the sizes of DLS are bigger. This phenomenon due to the DLS measures the sizes in the hydrated state (hydrodynamic size) whereas TEM and XRD measure the sizes in a solid form (dry state) [70]. Also, the homogeneity percentages and capping agents from fungal metabolites are two factors that interfere with particle size detection using DLS [71, 72]. In a recent study, the average particle size of phyto-synthesized gold nanoparticles was 15.1 and 18 nm using TEM and XRD respectively whereas the bigger size was obtained by DLS analysis which was 91.3 nm [55]. Also, the particle size of fungal-mediated biosynthesis of CuO-NPs was 18, 17.5, and 299.5 nm based on TEM, XRD, and DLS analysis respectively [21]. Also, the average sizes of CuO-NPs synthesized by leaf aqueous extract of *Enicostemma axillare* were 6.4 nm by TEM, 22.9 nm by XRD, and 88–307 nm by DLS analysis [70].

The DLS analysis give more information about the homogeneity percentages of CuO-NPs in colloidal solution via measuring of polydispersity index (PDI). This index was represented by values in the ranges of 0–1. The homogeneity of solution was increased or decreased at 0.4 ≤ PDI ≥ 0.4 respectively whereas the distribution of CuO-NPs in the colloidal solution became heterogenous at PDI value more than 1 [73]. In the current study, the PDI value of synthesized CuO-NPs was 0.362 which indicate the homogeneity of CuO-NPs in a colloidal solution. In a similar study, the PDI value of CuO-NPs synthesized by an aqueous extract of *E. axillare* was 0.782 [70].

#### ζ-potential Analysis

The tendency of NPs to flow in a solution when subjected to an electric field is known as electrokinetic or ζ-potential. It is a reliable and user-friendly technique for detecting the stability of NPs [74]. According to a guideline, the stability can be categorized according to the following criteria: high unstable at ζ values of ± 0–10 mV, relatively stable at ζ value in the ranges of ± 10–20mV, moderate stable at values of ± 20–30 mV, and high stable at ζ values greater than ± 30 mV [75]. In the current study, the ζ value of synthesized CuO-NPs was − 26.6 mV which indicates the high stability in the colloidal solution (Fig. 5B). This indicates the high stability of synthesized CuO-NPs, the analysis was carried out in a wide range of ζ-values (positive and negative values) to detect all charges on the NPs surface. The ζ-potential spectrum showed that all CuO-NPs have a negative charge, which indicate that the particles remain for-away from each other, and this prevent the agglomeration or aggregation with time [76]. Also, the presence of varied capping agent that cover the surface of NPs can be increase the stability as reported previously [77].

### Antimicrobial activity

**Fig. 6:**
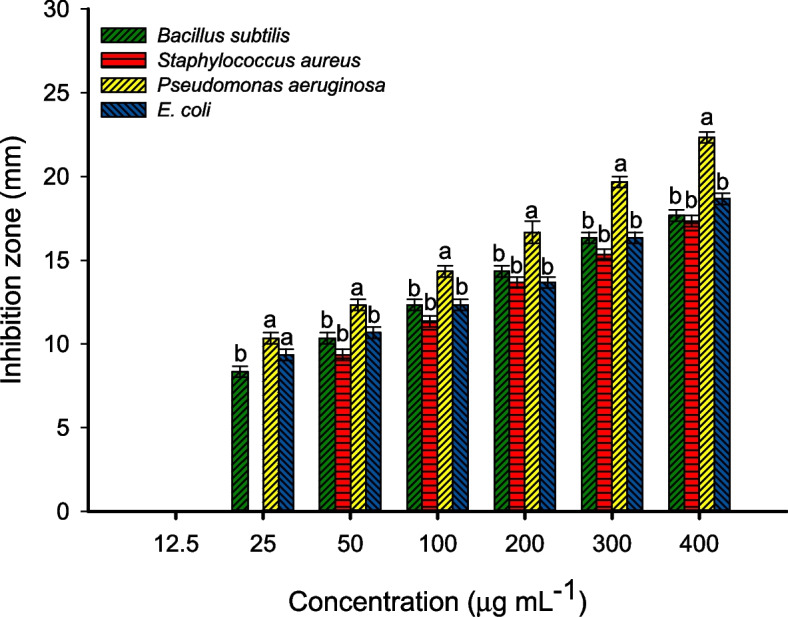
Antibacterial activity of CuO-NPs fabricated by endophytic fungal strain *A. terreus* BR.1 against Gram-positive bacteria (*B. subtilis and S. aureus*) and Gram-negative bacteria (*P. aeruginosa and E. coli*). Different letters at the same concentration indicate the significant values (*p ≤* 0.05)

**Fig. 7:**
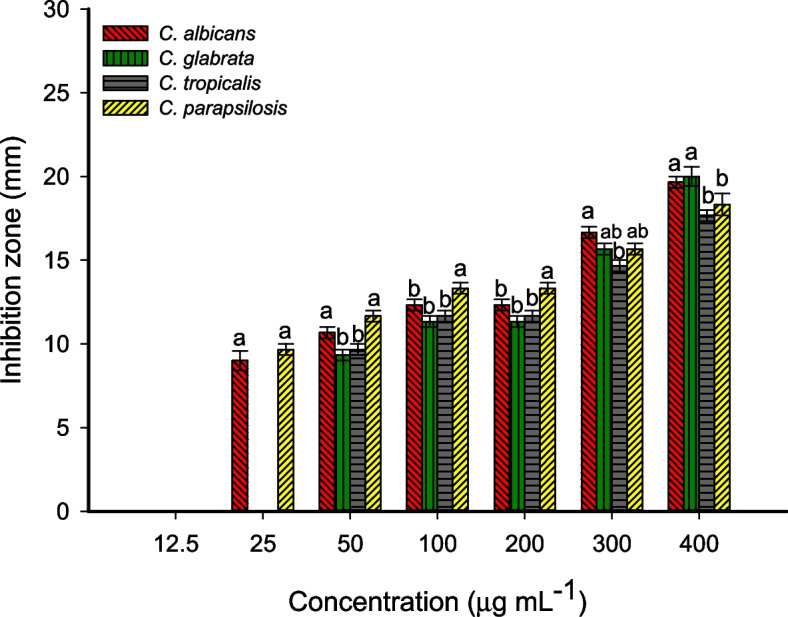
Anti-*Candida* activity of fungal synthesized CuO-NPs against various *Candida* spp. represented by *C. albicans, C. glabrata, C. tropicalis*, and *C. parapsilosis. *Different letters at the same concentration indicate the significant values (*p* ≤ 0.05)

The misuse of antibiotics and the spread of resistant antibiotic genes between different bacterial strains leads to the emergence of antibiotic-resistant bacteria causing thousands of deaths worldwide [78]. Therefore, the efforts are achieved for the synthesis of new active compounds based on a simple, fast, cost-effective, and environmentally friendly approach. Herein, the CuO-NPs fabricated by cell-free filtrate of endophytic fungal strain, *A. terreus* BR.1, were used to control the growth of various pathogenic microbes of Gram-positive bacteria including *Bacillus subtilis* and *Staphylococcus aureus*, Gram-negative bacteria including *E. coli* and *Pseudomonas aeruginosa*, and unicellular fungi represented by various strains of *Candida* (*C. albicans, C. glabrata, C. tropicalis*, and *C. parapsilosis*) by agar well diffusion method. Data analysis displayed that the activity of synthesized CuO-NPs toward various microbes was concentration-dependent manner, which is compatible with various published literature [10, 13, 49]. At high CuO-NPs concentration (400 µg mL^–1^), the zones of inhibitions (ZOIs) were 17.7 ± 0.6, 17.3 ± 0.6, 22.3 ± 0.5, and 18.7 ± 0.6 for *B. subtilis, S. aureus, P. aeruginosa*, and *E. coli* respectively (Fig. 6). Similarly, the highest clear zones against *Candida* spp. were achieved at a maximum concentration (400 µg mL^–1^) to be 19.7 ± 0.6, 20.0 ± 1, 17.7 ± 0.6, and 18.3 ± 1.3 mm for *C. albicans, C. glabrata, C. tropicalis*, and *C. parapsilosis* (Fig. 7). These activities were decreased at low concentrations to be (14.3 ± 0.6, 13.7 ± 0.6, 16.7 ± 1.2, and 13.7 ± 0.6 mm) and (12.3 ± 0.6, 11.3 ± 0.6, 11.6 ± 0.6, and 13.3 ± 0.6 mm) for (*B. subtilis, S. aureus, P. aeruginosa*, and *E. coli)* and (*C. albicans, C. glabrata, C. tropicalis*, and *C. parapsilosis*) respectively at 200 µg mL^–1^. In a similar study, the significant antibacterial and antifungal activity of green synthesized CuO-NPs toward *B. subtilis, S. aureus, P. aeruginosa, E. coli, Acinetobacter* sp., *Sphingobacterium* sp., *Ochrobactrum* sp. *Aspergillus* sp., *Trichoderma* sp., *Meyerozyma* sp., and *Fusarium* sp. were accomplished at high concentrations (170 ppm) compared to the activity at low concentrations (100 and 50 ppm) [65]. Also, the maximum ZOIs due to the treatment of *Mycobacterium tuberculosis, S. aureus, Klebsiella pneumoniae, E. coli, Corynebacterium diphtheriae, Proteus mirabilis*, and *Streptococcus pyogenes* with 100 µg mL^–1^ were 54 ± 1.9, 30.3 ± 1.3, 30.5 ± 1.5, 36.2 ± 1.7, 35.8 ± 2.0, 34.7 ± 1.8, and 22.5 ± 1.1 mm respectively [31]. These ZOIs were reduced by decreasing the concentrations. Moreover, the highest antifungal activity of biosynthesized CuO-NPs against *C. albicans, C. glabrata, A. flavus*, and *Microsporum canis* was achieved at 100 µg mL^–1^ and decreased at 50 and 10 µg mL^–1^ [31].

The treatment strategy was chosen based on a reliable MIC value which is essential for effective infection prevention and control [79]. In the current study, the synthesized CuO-NPs showed antimicrobial activity with a low MIC value. As shown, the MIC values for different pathogenic bacteria were 25 µg mL^–1^ for *B. subtilis, P. aeruginosa*, and *E. coli* with inhibition zones in the ranges of 8.3 ± 0.6–10.3 ± 0.6 mm. Whereas the MIC value for Gram + *S. aureus* was 50 µg mL^–1^ with an inhibition zone of 9.3 ± 0.5 mm (Fig. 6). Similarly, the MIC value for *C. albicans, C. glabrata*, and *C. tropicalis* was 50 µg mL^–1^, whereas *C. parapsilosis* has a MIC value of 25 µg mL^–1^ (Fig. 7). Recently, the MIC values of CuO-NPs fabricated by endophytic bacterial strain *Brevibacillus brevis* were 6.25, 12.6, 6.25, and 50 µg mL^–1^ for *C. glabrata, C. tropicalis, C. albicans*, and *C. parapsilosis* respectively [49]. Incompatible with the obtained results, CuO-NPs synthesized by leaves aqueous extract of *Aerva javanica* showed MIC values against various strains of *Candida* in the ranges of 160–300 µg mL^–1^ [3]. Also, the chemically synthesized CuO-NPs showed MIC value against *C. albicans* with a value of 400 µg mL^–1^ [80]. The difference in MIC value against pathogenic microbes could be attributed to the synthesis method, sizes, shapes, aggregation percentages, and surface charge [81, 82].

As shown, the highest activity of biosynthesized CuO-NPs was noticed against Gram-negative bacteria, especially *P. aeruginosa.* This finding could be related to the bacterial cell wall structure which in Gram-negative bacteria is composed of a thin layer of peptidoglycans and hence CuO-NPs can penetrate through it easily. In contrast, the cell wall of Gram-positive bacteria containing thick peptidoglycan layers that delay or prevent the penetration of active compounds [83, 84]. The activity of CuO-NPs to inhibit the pathogenic microbes depends on different factors such as incubation times of NPs with organisms, concentrations, incubation temperature, agglomeration percentages, and structure of surface area [85]. The production of toxic ions which in the current study are Cu^2+^ after the breakdown of NPs inside the bacterial cells is considered the main mechanism for the inhibitory action of CuO-NPs. These liberated toxic ions react with the thiol group of protein and hence inhibit their function [86]. Also, the accumulation of these ions can be destroying the selective permeability function of the cytoplasmic membrane [87]. Another inhibitory mechanism of CuO-NPs is their activity to a transcript of different oxidative stress genes that enhance the production of reactive oxygen species (ROS). The ROS has deleterious effects on the cell components such as proteins, ribosomes, nucleic acids, amino acids, and membrane components that ultimately to cell death [88–90]. Moreover, CuO-NPs have negative impacts on the ergosterol synthesis pathway and hence destroy the sterol profile in Candida cell wall leading to cell death [91, 92].

### In-vitro Cytotoxicity and biocompatibility assay

One of the biggest threats to global health that has not yet been addressed is cancer development infection. Recently, several active substances including NPs demonstrated strong activity against a variety of cancer cells, allowing them to be included in cancer therapy or employed as carriers or delivery systems for medications used to treat cancer [3, 93]. The nanomaterial-based cancer treatment, especially those fabricated by green approaches, are widely investigated because of their biocompatibility, permeability, easy scale-up, biodegradability, safe to normal cells at specific concentrations [14, 94]. The biocompatibility of nanomaterials against normal cells is a crucial factor that should be investigated before being used as an anti-cancer agent. The MTT assay method is accurate, sensitive, colorimetric, and possesses the ability to evaluate metabolic cell activities by calculating the viable cells after treatment with active substances [36]. Therefore, in the current study, the activity of fungal-mediated CuO-NPs against two normal cell lines namely Vero and Wi38, and two cancer cell lines designated as MCF7 and PC3 were investigated using the MTT assay method. As shown, the biosynthesized CuO-NPs have a concentration-dependent toxic effect on normal and cancer cell lines. The obtained finding is compatible with those reported by Amin et al., who reported that the cell viability of Neuroblastoma cell lines was dependent on the concentration of green synthesized CuO-NPs treatment, the viability increased at low concentrations and decrease at high concentrations [3]. Also, CuO-NPs synthesized by endophytic fungal strain *A. terreus* exhibit anticancer activity against HT-29 (colon cancer cell lines) in a concentration-dependent manner [14].

**Fig. 8:**
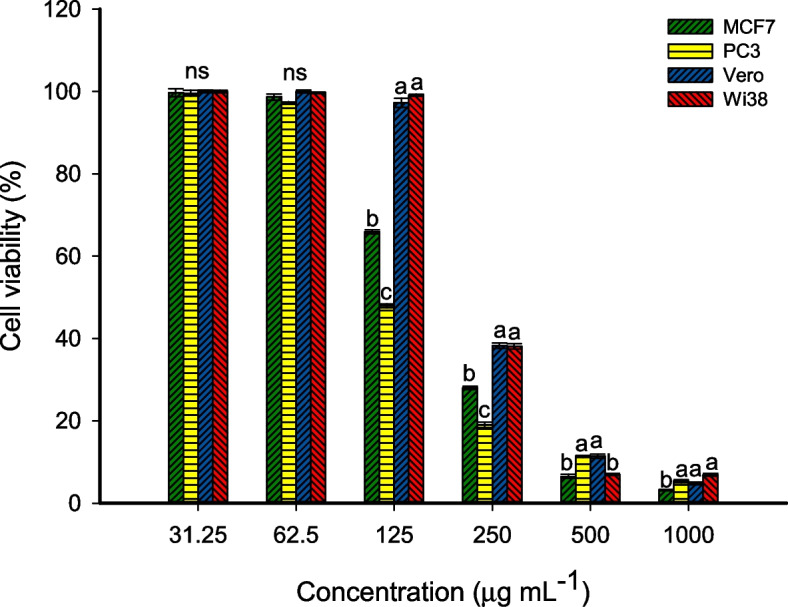
Cell viability assay using MTT assay method of green synthesized CuO-NPs treated normal cells (Wi38 and Vero cell lines) and cancer cells (MCF7 and PC3). ns is meaning the data at this concentration are not significant. Different letters at the same concentration indicate the significant values (*p* ≤ 0.05)

Data analysis showed that the lowest cell viability was attained at high concentrations of 100 and 500 µg mL^–1^ for normal and cancer cell lines. As shown, the cell viability percentages were (4.8 ± 0.5, 6.9 ± 0.6, 3.2 ± 0.3, and 5.3 ± 0.5%) and (11.4 ± 0.9, 7.0 ± 0.4, 6.5 ± 0.5, and 11.3 ± 0.4%) at concentrations of 100 and 500 µg mL^–1^ for Vero, Wi38, MCF7, and PC3 cell lines respectively after 48 h of incubation (Fig. 8). The cell viability increased at low concentrations. For instance, the viability of normal cells, Vero and Wi38 with percentages of 97.2 ± 1.9% and 99.1 ± 0.5% were attained at a concentration of 125 µg mL^–1^, whereas the 65.8 ± 0.8% and 47.9 ± 0.7% viability of cancer cell lines, MCF7 and PC3 were attained at the same concentration (125 µg mL^–1^). As seen, the death of normal cells at low concentrations was less than the percentages of cancer cell death at the same concentrations. This phenomenon can be benefited by using such low concentrations to be targeting the cancer cells without affecting normal cells. Similarly, the cell viability of breast cancer cell lines (T47D) and normal cell lines (HFB4) were highly decreased at a maximum concentration of CuO-NPs fabricated by endophytic strain to be 3.7 and 5.8% respectively. These percentages were increased to 49% for T47D and 92% for HFB4 after 48 h of incubation [49]. Also, the cell viability of two cancer cell lines, MCF-7 and AMJ-13 was 19.4 and 26.3% at the maximum concentration used of green synthesized CuO-NPs (100 µg mL^–1^) after an incubation period of 48 h [95]. Several published literatures reported that the cytotoxic efficacy of green synthesized CuO-NPs toward cancer cell lines was higher than NPs fabricated by physical and chemical methods [14, 95, 96].

The IC_50_ values (the concentration of NPs causing 50% cell viability) were detected. Data analysis showed that the IC_50_ value of normal cell lines was 220.6 ± 3.7 µg mL^–1^ for Vero cell lines and 229.5 ± 2.1 µg mL^–1^ for Wi38 cell lines. These values increased with percentages of 30–45% as compared with IC_50_ for cancer cell lines. Data showed that the IC50 of cancerous cell lines was 159.2 ± 4.5 µg mL^–1^ for MCF7 and 116.2 ± 3.6 µg mL^–1^ for PC3. In a similar study, the IC_50_ value of CuO-NPs synthesized by the action of metabolites secreted by fungal strain *A. terreus* was 192.2 µg mL^–1^ for the normal cell line (Wi38) and 96.3 µg mL^–1^ for cancer cell line (Caco-2) [97]. The obtained data opens the window to incorporate green synthesized CuO-NPs in cancer cell therapy with a concentration that is safe for normal cell lines.

There are several mechanisms that can explain the anticancer activity of CuO-NPs such as apoptosis, ROS production, autophagy, and antioxidant activity, blocking the normal mammalian cell cycles. All these mechanisms depend on the synthesis approach (chemical, physical, and biological), the source of cells, and concentrations [98, 99]. Apoptosis is defined as cell death due to cellular stresses and nuclear material damage controlled by a group of endoprotease enzymes (caspases) through intrinsic and extrinsic pathways [100]. The expression of these pathways can be upregulated as a result of CuO-NPs treatment, ultimately enhancing apoptosis [101]. The reaction of CuO-NPs with mitochondria enhances the production of ROS such as ^–^O_2_, H_2_O_2_, ^•^OH which have deleterious effects on DNA, proteins, amino acids, and cell membrane [102]. The production percentages of ROS in cancer cells are more than normal cells due to extreme metabolic activity and can benefit from this finding in cancer therapy using NPs [98]. Also, the liberation of toxic ions (Cu^2+^) after the reaction of CuO-NPs with cells leads to the destruction of macromolecules inside the cells, besides enhancing the production of ROS [24].

### Antioxidant activity

**Fig. 9:**
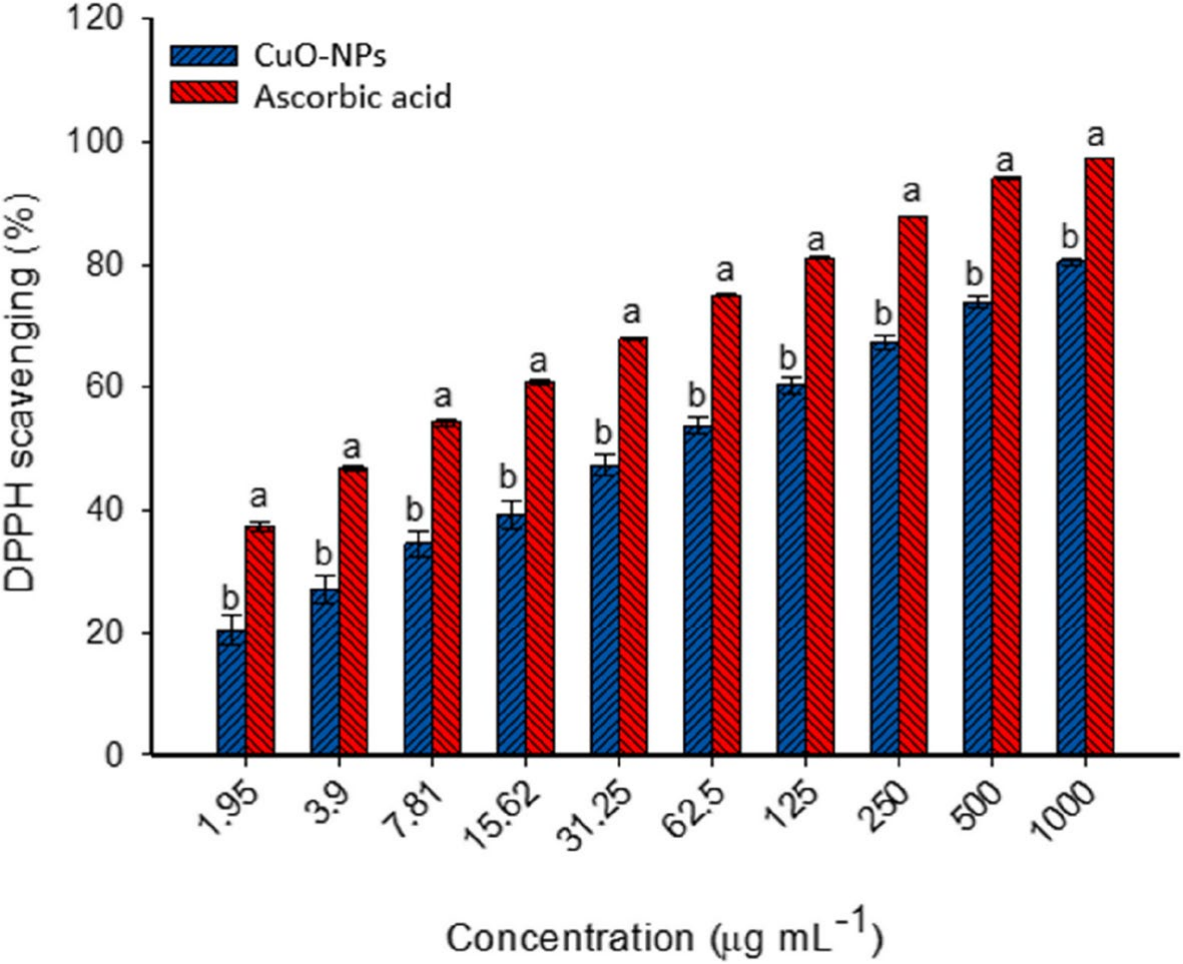
Antioxidant activity of fungal synthesized CuO-NPs compared to ascorbic acid (control). Different letters at the same concentration indicate the significant values (*p* ≤ 0.05)

Recently, the investigation of the antioxidant activity of NPs to be incorporated into the biological systems is a critical point. Free radicals are produced in different biological systems due to the interaction between molecular oxygen and biomolecules [17]. These radicals contain one or more unpaired electrons and are characterized by extreme unstable causing damage to biological molecules through the extraction of electrons from them to become stable [15]. The antioxidant activity of synthetic and natural compounds has been due to different mechanisms such as peroxides decomposition, blocking of chain initiation, preventing of abstraction of molecular oxygen, scavenging of free radicals, and reductive capacity [103]. The scavenging DPPH assay method is the most common method for investigating the antioxidant property of new active compounds. In the current study, the antioxidant activity of green synthesized CuO-NPs was studied using the DPPH scavenging method. As shown, the antioxidant property of CuO-NPs was directly proportional to CuO-NPs concentrations (concentration-dependent manner) (Fig. 9). This finding is compatible with published literature on the antioxidant activity of green synthesized CuO-NPs [15, 17]. At high concentration (1000 µg mL^–1^), the green synthesized CuO-NPs showed scavenging activity with percentages of 80.5 ± 1.2% compared to ascorbic acid (control) which recorded the scavenging activity of 97.3 ± 0.2% (Fig. 9). These percentages were decreased at low concentrations. For instance, the scavenging percentages were 67.5 ± 2.1% and 87.7 ± 0.1% for CuO-NPs and ascorbic acid respectively at a concentration of 250 µg mL^–1^. At the lowest concentration (1.95 µg mL^–1^), the synthesized CuO-NPs still possess antioxidant activity with a value of 20.4 ± 4.2%. Similarly, the CuO-NPs fabricated by aqueous extract of heartwood of *Suaeda maritima* showed antioxidant activity detected by DPPH scavenging methods with a value of 83.9% at a concentration of 40 µg mL^–1^ compared to ascorbic acid (95.2 µg mL^–1^) at the same concentration [16]. The authors reported that the scavenging activity decreased with low concentrations to recorded values of 3.5% and 6.5% for CuO-NPs and ascorbic acid respectively at a concentration of 5 µg mL^–1^. Also, CuO-NPs fabricated by plant extract of *Camellia sinensis* and *Prunus africana* showed antioxidant activity with percentages of 28.8% and 28.5% respectively at a concentration of 300 µg mL^–1^ compared to the value of ascorbic acid (70.8%) at the same concentration [1]. These percentages were decreased to 17.5% and 16.9% respectively at a concentration of 50 µg mL^–1^ compared to the control (23.4%).

The effective concentration of CuO-NPs needed for scavenging 50% of free radicals (EC_50_) was detected. As shown, the EC_50_ value of fungal-based CuO-NPs was 54.9 ± 1.4 µg mL^–1^ compared to the EC_50_ value of ascorbic acid (6.7 ± 1.5 µg mL^–1^). In a similar study, the EC50 of synthesized CuO-NPs was 28.1 µg mL^–1^ compared to ascorbic acid (23.7 µg mL^–1^) [16].

The protection of cells from damage caused by unstable molecules or free radicals that are produced under various stresses like contaminants, pathogens, radioactive chemicals, poisons, etc. is achieved by using antioxidant compounds or free radical scavengers [20]. Various diseases such as heart attack, rheumatism, leukemia, immunological dysfunction, respiratory failure, neurological diseases (such as Parkinson’s, Alzheimer’s, multiple sclerosis, and memory loss), renal failure, uremia, proteinuria, and metabolic problems are considered the main symptoms for free radicals [104]. Natural products (such as ascorbic acid, flavonoids, tannins, phenolic compounds, and phytoestrogens), as well as synthetic compounds (such as Triazines and nanomaterials), are used as scavengers for free radicals [17, 105]. Rehana et al. [15] reported that the antioxidant activity of synthesized CuO-NPs is higher compared to those synthesized by a chemical method due to the presence of fungal secondary metabolites such as carbohydrates and phenols used for the fabrication of CuO-NPs. The antioxidant mechanism of metal oxide nanoparticles could be due to the connecting of transition metal ions with free radicals leading to improve scavenging activity [1].

## Conclusion

In the current study, CuO-NPs were successfully synthesized using biomass filtrate of endophytic fungal strain which was identified using traditional and molecular identification as *Aspergillus terreus* BR.1. The synthesized CuO-NPs showed maximum SPR at a wavenumber of 280 nm which signify to the SPR of CuO. Moreover, spherical shapes with sizes of 15–55 nm and crystalline structures were improved by TEM and XRD. The Cu and O ions represented the main components of the as-formed material. The surface charge of synthesized CuO-NPs was − 26.6 mV as detected by the zeta potential value which indicates high stability of the synthesized compound in colloidal solution. Interestingly, green synthesized CuO-NPs exhibit promising activity against pathogenic Gram-positive bacteria, Gram-negative bacteria, and various strains of *Candida* as unicellular fungi. Also, the synthesized CuO-NPs showed high biocompatibility toward normal cell lines and *in-vitro* cytotoxicity against cancer cells with low IC_50_ value. The scavenger activity was estimated by DPPH methods which reveals the scavenging activity at low concentrations. Overall, the endophytic fungal strains especially those isolated from medicinal plants have the capacity to fabricate CuO-NPs with promising biomedical applications due to high metabolites production which is used to cap and stabilize the final product.

## Data Availability

The datasets generated and/or analyzed during the current study are available upon request from the corresponding authors. The sequence of the obtained endophytic fungal strain was deposited in GenBank under web link of https://www.ncbi.nlm.nih.gov/nuccore/OP471233.
